# Comparison of protocols with respiratory-gated (4D) motion compensation in PET/CT: open-source package for quantification of phantom image quality

**DOI:** 10.1186/s40658-022-00509-4

**Published:** 2022-11-17

**Authors:** Andrea Martinez-Movilla, Michael Mix, Irene Torres-Espallardo, Elena Teijeiro, Pilar Bello, Dimos Baltas, Luis Martí-Bonmatí, Montserrat Carles

**Affiliations:** 1 Biomedical Imaging Research Group (GIBI230-PREBI) and Imaging La Fe node at Distributed Network for Biomedical Imaging (ReDIB), Unique Scientific and Technical Infrastructures (ICTS), La Fe Health Research Institute, Valencia, Spain; 2 Department of Nuclear Medicine, University Medical Center Freiburg, Faculty of Medicine, 79106 Freiburg, Germany; 3 Department of Nuclear Medicine, Medical Imaging Clinical Area, La Fe University and Polytechnic Hospital, 46026 Valencia, Spain; 4 Department of Radiation Oncology, Division of Medical Physics, University Medical Center Freiburg, Faculty of Medicine, 79106 Freiburg, Germany; 5 German Cancer Consortium (DKTK), Partner Site Freiburg, Germany

**Keywords:** Gated 4D PET/CT, Motion compensation, Experimental phantoms, Open-source package, Quality assurance

## Abstract

**Background:**

Patient’s breathing affects the quality of chest images acquired with positron emission tomography/computed tomography (PET/CT) studies. Movement correction is required to optimize PET quantification in clinical settings. We present a reproducible methodology to compare the impact of different movement compensation protocols on PET image quality. Static phantom images were set as reference values, and recovery coefficients (RCs) were calculated from motion compensated images for the phantoms in respiratory movement. Image quality was evaluated in terms of: (1) volume accuracy (VA) with the NEMA phantom; (2) concentration accuracy (CA) by six refillable inserts within the electron density CIRS phantom; and (3) spatial resolution (R) with the Jaszczak phantom. Three different respiratory patterns were applied to the phantoms. We developed an open-source package to automatically analyze VA, CA and R. We compared 10 different movement compensation protocols available in the Philips Gemini TF-64 PET/CT (4-, 6-, 8- and 10-time bins, 20%-, 30%-, 40%-window width in Inhale and Exhale).

**Results:**

The homemade package provided RC values for VA, CA and R of 102 PET images in less than 5 min. Results of the comparison of the 10 different protocols demonstrated the feasibility of the proposed method for quantifying the variations observed qualitatively. Overall, prospective protocols showed better motion compensation than retrospective. The best performance was obtained for the protocol Exhale 30% (0.3 s after maximum Exhale position and window width of 30%) with RC$_{VA}=1.6\pm 1.3$, RC$_{CA}=0.90\pm 0.09$ and RC$_{R}=0.6 \pm 0.4$. Among retrospective protocols, 8 Phase protocol showed the best performance.

**Conclusion:**

We provided an open-source package able to automatically evaluate the impact of motion compensation methods on PET image quality. A setup based on commonly available experimental phantoms is recommended. Its application for the comparison of 10 time-based approaches showed that Exhale 30% protocol had the best performance. The proposed framework is not specific to the phantoms and protocols presented on this study.

## Background

Positron emission tomography/computed tomography (PET/CT) acquisition with $^{18}$F-fluorodeoxyglucose ($^{18}$F-FDG) as radiotracer has been proven to be of the utmost importance in the assessment of tumors [1], in the planning of patient treatments [2] and guiding biopsy [3]. As PET acquisition times are much longer than the respiratory cycle, patient’s breathing causes respiratory motion, blurring the spatial distribution of the radiotracer’s concentration and hindering the delineation of tumors [4] in the thoracic area. Moreover, the expansion and compression of the lungs while breathing affects the computation of attenuation coefficients and influences the attenuation correction of PET images [5]. Consequently, respiratory motion leads to an inaccurate quantification of tumor size and standardized uptake value (SUV).

Commercially available PET/CT systems have traditionally monitored respiratory cycle through the use of external hardware [6–8]. PET/CT manufacturers have recently started implementing data-driven motion correction [5, 9–11], for which the respiratory signal is derived from the PET list-mode data, and consequently, no external devices are required. Independently of the method employed to identify the respiratory pattern, 3D images are then reconstructed based on subdivisions of the patient’s breathing cycle. Two main groups could be distinguished: prospective protocols, where we specify beforehand in which parts of the breathing cycle data are acquired, and retrospective protocols, where data are acquired through all the PET acquisition time but then each breathing cycle is split in the desired number of frames. Breathing cycles could be divided based on time or on amplitude criteria [7]. The use of these protocols reduces image smearing and improves definition of the lesion volume and the quantification of lesion activity [12]. Several respiratory-gated PET/CT acquisitions have been proven useful in clinical settings [5], with a few comparison studies [13–15]. Most recently proposed techniques [16, 17] additionally combined the resulted 3D images phases by choosing one reference frame and registering the other frames with respect to it. The resulted unique motion-corrected frame preserves all acquisition counts, and therefore, image noise has been proved to be reduced. The use of mathematical models to compensate the respiratory motion has been also investigated [18]. In order to establish the best approach, a standard procedure for the comparison of the different compensation motion methods is desirable.

Standardization and validation of the different motion compensation techniques are a prerequisite before their clinical use [15]. Phantoms could be employed for the standardization of respiratory movement correction protocols. In contrast to patient’s examinations, they allow replicability of studies. They also provide nominal values for density, volume and activity concentration, against which image measurements can be contrasted. In addition, phantoms can undergo long PET/CT studies and as many PET/CT scans as necessary with no safety limitations.

The number of tools for image processing has significantly increased in the last years and could be divided in two main subgroups: processing packages oriented to clinical imaging or processing packages oriented to imaging system quality control. Within the first group, there are powerful tools such as Nipype, and Nipype-based software adaptations (such as APPIAN and Pypes), focused on PET/MR neuroimaging. Most of the PET processing packages of the second subgroup are commercially available and/or exclusively designed for specific phantoms [19–22]. From our knowledge, no packages oriented to PET motion compensation quality control exist neither as open-source format, nor in the market.

In this study, we present a method for the comparison of the quality of motion compensation protocols, by using experimental phantoms. An open-source package for automatic analysis was developed with this purpose. PET image quality has been evaluated in terms of resolution, volume accuracy and the accuracy in the estimation of activity concentration. As a proof of concept, the proposed setup and analysis were applied for the comparison of the movement compensation protocols available in a PET/CT system. Furthermore, in order to demonstrate the general applicability to other phantoms and to other systems, an additional comparison of two motion compensation protocols available in a PET/MR system was carried out by using a different phantom.

## Materials and methods

### Experimental phantoms

**Fig. 1:**
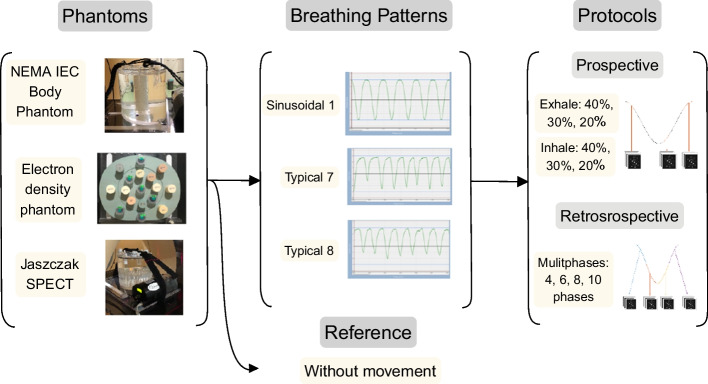
Materials and methods used for the comparison of the different protocols for respiratory motion compensation

This study employed three different phantoms, using each of them to assess a different image quality metric, as shown in Fig. 1.

#### NEMA phantom

**Fig. 2:**
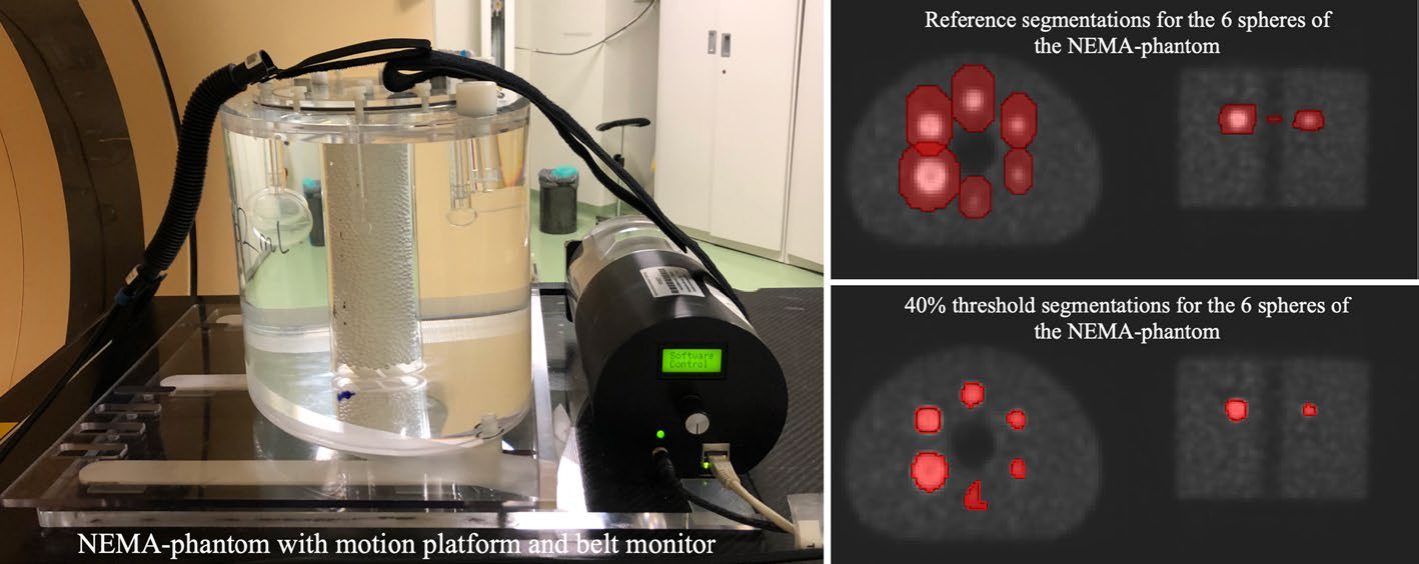
Setup employed for volume quantification. Axial, coronal an sagittal view of the PET static image of NEMA phantom and examples of the references and the automatic segmentations

The volume quantification phantom (NEMA IEC body phantom, Fig. 2) consists of a 9.7 l phantom and an insert with six hollow spheres of volumes 0.52 ml, 1.15 ml, 2.57 ml, 5.57 ml, 11.49 ml and 26.2 ml. The average activity concentration was 80 kBq ml$^{-1}$ for the spheres and 15 kBq ml$^{-1}$ for the background within body compartment.

#### CIRS phantom

**Fig. 3:**
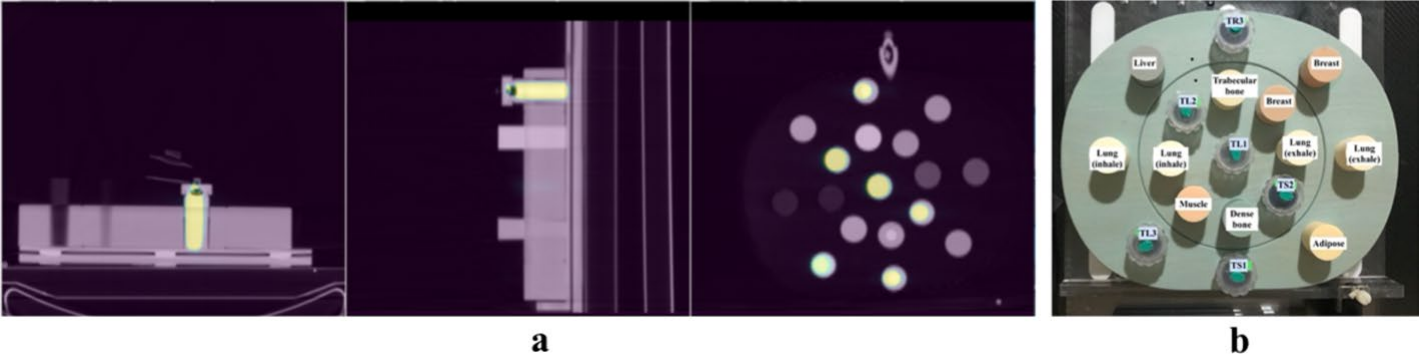
**a** Axial, sagittal and coronal slices of static PET/CT image of the CIRS phantom with the six inserts filled with activity. **b** CIRS phantom with the inserts’ nomenclature

For activity quantification, the electron density phantom (CIRS phantom) was used. It consists of two plastic disks (Plastic Water®) with 17 holes having 30 mm of diameter (Fig. 3). These holes can be filled with inserts which replicate different densities observed in the clinical environment (Table 1). In addition, six homemade refillable inserts were placed within the phantom, three with 33 ml volume (TL) and three of 11 ml (TS). The inserts had an average activity concentration of 900 kBq ml$^{-1}$.

**Table 1:** Densities for the inserts of the CIRS phantom

Description	Physical density (g$/{cm}^3$ )	Electron density (10$^{23}$ e$^-/{cm}^3$)
Lung (Inhale)	0.205	0.668
Lung (Exhale)	0.507	1.658
Breast (50% Gland/50% Adipose)	0.99	3.261
Trabecular bone (200 mg/cc HA)	1.16	3.730
Liver	1.07	3.516
Muscle	1.06	3.483
Adipose	0.96	3.171
Dense bone (800 mg/cc HA)	1.53	4.862
Dense bone (1250 mg/cc HA)	1.82	5.663
Water–liquid	1.00	3.340

#### Jaszczak phantom

For the spatial resolution quantification, the Flangeless Esser PET Phantom (Data Spectrum) phantom was employed. It consists of a cylindrical body phantom of 6.1 l, including PMMA inserts. It contains solid rods arranged into sectors, with diameters of 4.8 mm, 6.4 mm, 7.9 mm, 9.5 mm, 11.1 mm and 12.7 mm. The average activity concentration was 7 kBq ml$^{-1}$.

#### Respiratory motion simulation

**Fig. 4:**
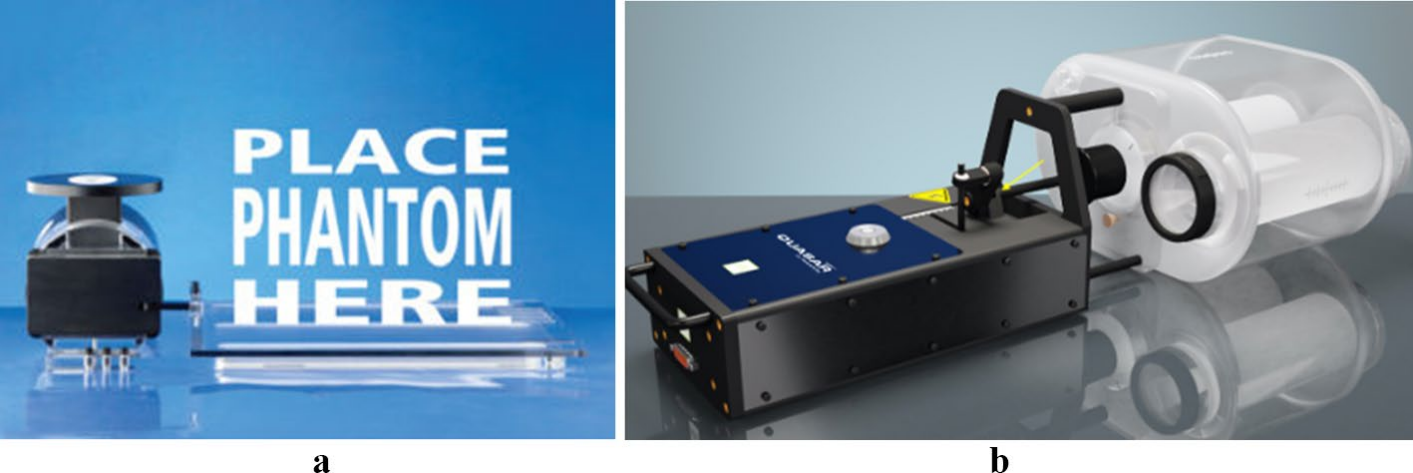
**a** QUASAR$^\mathrm{{TM}}$ Respiratory Motion Platform. Image taken from [23], **b** QUASAR$^{TM}$ MRI$^{4D}$ Motion Phantom. Image taken from [24]

The breathing cycle was simulated with the QUASAR$^\mathrm{{TM}}$ Respiratory Motion Platform [23] for PET/CT and with the QUASAR$^\mathrm{{TM}}$ MRI$^{4D}$ Motion Phantom for PET/MR, both by the manufacturer Modus Medical Devices [24]. The first model consists on a platform where a phantom can be placed (Fig. 4a). Consequently, the movement of the platform translates on a movement of the phantom. For our PET/CT setup, the phantoms were NEMA, CIRS and Jaszczak. The QUASAR$^\mathrm{{TM}}$ MRI$^{4D}$ Motion Phantom is compatible with the presence of magnetic fields, and the movement is applied to the specific designed phantom, which consists of a refillable sphere within a cylindrical lung insert placed in an oval body container (Fig. 4b).

The phantoms were driven in the superior–inferior direction, with periods between 4 and 5 s and different respiratory patterns provided by the manufacturer. For the evaluation of PET/CT, the patterns were: Sinusoidal 1, commonly employed as approach of the clinical case [6] and Typical 7 and Typical 8, which are real breathing patterns of patients from the category typical [25], where typical refers to 60% of the population and exhibits a regular breathing. The mean peak-to-peak amplitude was 28 mm for Sinusoidal 1, 25 mm for Typical 7 and 23 mm for Typical 8. Hysteresis with an amplitude of 3 mm was applied to Typical 7 and Typical 8 patterns [26]. For PET/MR evaluation 4 patterns were applied: Sinusoidal, Sinusoidal with rotation simultaneously, Typical 7 and Irregular 4, all of them provided by the manufacturer.

### Equipment

The PET/CT hybrid system Gemini TF-64 (Philips Medical Systems, Cleveland, Ohio, USA) [27] was used to acquire static and respiratory-gated PET/CT image data. The transverse spatial resolution at 1 cm from the central axis of the scanner was 4.7 mm, the temporal resolution was 526 picoseconds and the average energy resolution was 11.8%. The reconstruction method for all scans was LOR-based ordered-subset iterative time-of-flight algorithm using spherical coordinates (BLOB-OS-TF) with two iterations and 33 subsets. For static PET total acquisition time was 11 min and resulting image had a voxel size of $2\times 2 \times 2\,{\text {mm}}^3$. For movement correction protocols, total acquisition time was 14 min and resulting image had a voxel size of $4\times 4 \times 4\,{\text {mm}}^3$. To monitor and record the platform movement, the breathing pattern belt monitor (Bellows device with Mayo Clinic Respiratory Feedback System) was used. It was connected to the PET/CT system and fastened around the phantom and the platform.

The SIGNA PET/MR (GE Medical systems, Waukesha, Wisconsin, USA) [28] was used for the experimental validation of the general applicability of the proposed workflow. The radial, tangential and axial spatial resolutions from the central axis of the scanner were 4.4, 4.1 and 5.3 mm, respectively. PET total acquisition time was 12 min, and resulting image had a voxel size of $2.34\times 2.34\times 2.78\,{\text {mm}}^3$.

### Motion compensation protocols

The protocols provided by the PET/CT and PET/MR systems used were time-based protocols. (The respiratory signal in each breath cycle was divided in terms of time.) The movement correction protocols could be divided into prospective and retrospective protocols. The reference image (static protocol) resulted from imaging phantoms, when no movement was applied.

*Prospective* The PET/CT system provides two types of prospective protocols: Inhale_max_ and Exhale max, where the acquisition is done 280 ms after the patient’s inhalation or exhalation, respectively. For each protocol, we made three reconstructions: 20%, 30% and 40% of breathing cycle duration. For the PET/MR system, we evaluated the Exhale protocol (Q.Static-50%) with two different windows (30% and 15%) and with and without rejection of the irregular breathing cycles obtained during acquisition (with and without trigger). Irregular breathing cycles are defined as cycles shorter or longer than the average cycle duration by a relative deviation predefined by the user (12% for Sinusoidal, 25% for Typical 7 and 50% for Irregular 4).

*Retrospective* In the retrospective protocol, PET acquisition was reconstructed in the number *N* of phases desired; that is, each breathing cycle (defined between two consecutive maximums of amplitude) was divided in *N* equal time intervals. *N* final images were derived from the reconstruction of the data corresponding to each phase. Concretely, 4 retrospective protocols, with 4, 6, 8 and 10 Phases, were evaluated.

### Evaluation of image quality

#### Volume quantification

In the volume quantification analysis, for each sphere of the NEMA phantom the 40% threshold segmentation method with a region growing algorithm was applied [29, 30]. The segmentations were automatically generated for all images, from the set of 6 segmentations manually performed by the user (reference segmentations in Fig. 2). Detailed information of the workflow for automation can be found in the package documentation.

To evaluate volume quantification accuracy, the volume recovery coefficient $\left( RC=\frac{V_{mov}}{V_{static}}\right)$ was defined. It compares the volume segmented on the PET image of the phantom without movement (static) with respect to the volume segmented on the PET images, obtained when motion compensation protocols were applied to the data acquired with the phantom following a respiratory pattern.

#### Activity concentration quantification

For the quantification of activity concentration, equal-diameter cylindrical segmentations were manually defined within the 6 refillable inserts of the CIRS phantom. For all motion compensated images, the segmentations were automatically generated from the set of 6 segmentations performed by the user on the static image. Detailed information of the workflow for automation can be found in the package documentation.

For each segmentation, the mean activity concentration (C) was computed on each motion compensated PET image. The results were compared to the static image by computing the activity concentration recovery coefficient $\left( RC= \frac{C_{mov}}{C_{static}}\right)$. For RC computation, the radioactive decay was compensated in the comparison of activity concentrations.

#### Spatial resolution quantification

**Fig. 5:**
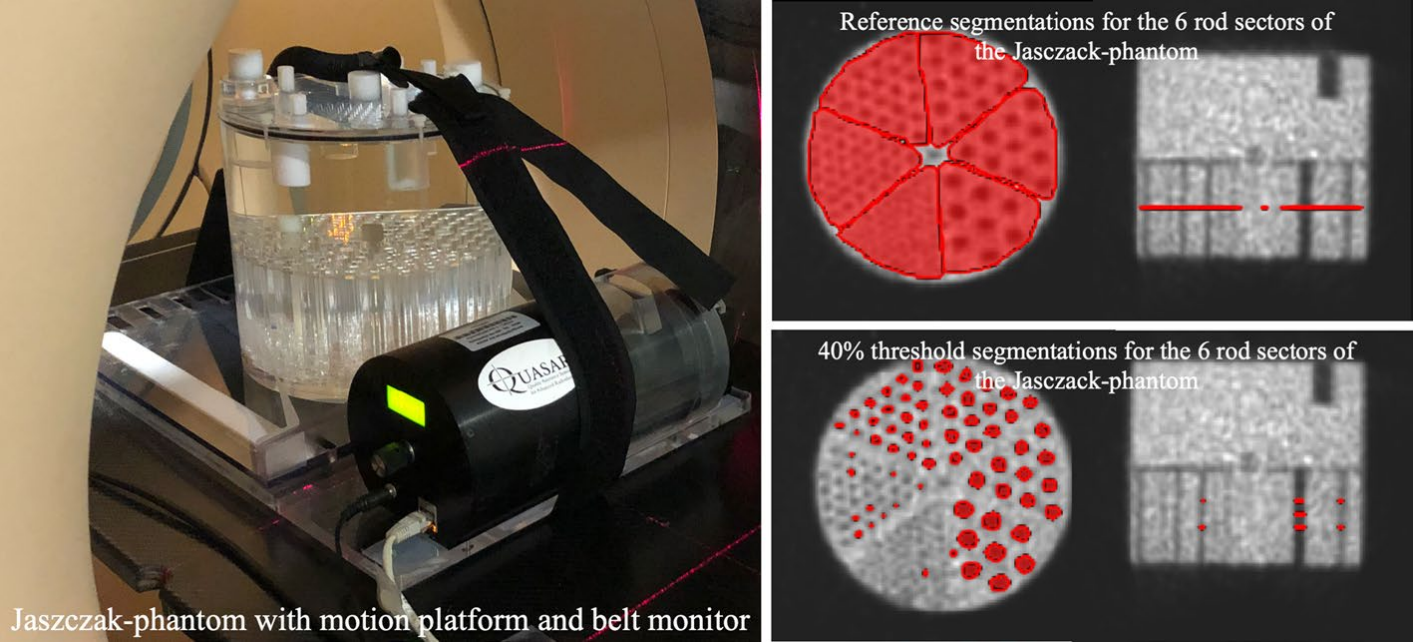
Setup employed for spatial resolution quantification. Axial, coronal and sagittal slices of the PET static image of Jaszczak phantom and examples of the references and the automatic segmentations

The aim of this part of the analysis was to evaluate the capability of the system to differentiate between structures and display them as separate entities on the image. In this study, we refer to this ability by the term resolution. To evaluate spatial resolution, triangular segmentations surrounding each rod sector on the Jaszczak phantom were required from the user (reference segmentation in Fig. 4) on three slices of the static PET image. In each triangular segmentation, a threshold of 40% of maximum was applied. Voxels with intensity lower than 40% of maximum, background (Bg), corresponded to the rods (see segmentation in Fig. 5). The segmentations were automatically generated for all images. Detailed information of the workflow for automation can be found in the package documentation.

For each segmentation, we calculated the recovery coefficients of the number of rods $\left( RC= \frac{Rod_{mov}}{Rod_{static}}\right)$. In addition, the contrast of each sector was computed $\left( Contrast=\frac{C_{FDG}}{C_{Bg}}\right)$.

#### Statistical analysis

The Wilcoxon signed rank test (WSRT) and the Bland–Altman (BA) percentage plot analysis were used to assess whether the performance of movement correction protocols was equivalent or not. The *p* value for significant difference criteria has been adjusted by using Bonferroni correction. For the BA plots, the criterion for significant bias was 95% coincidence interval (CI) of the mean differences relative to the mean must comprise zero [31]. All the statistics were performed using Python.

## Results

### Open-source package validation

**Fig. 6:**
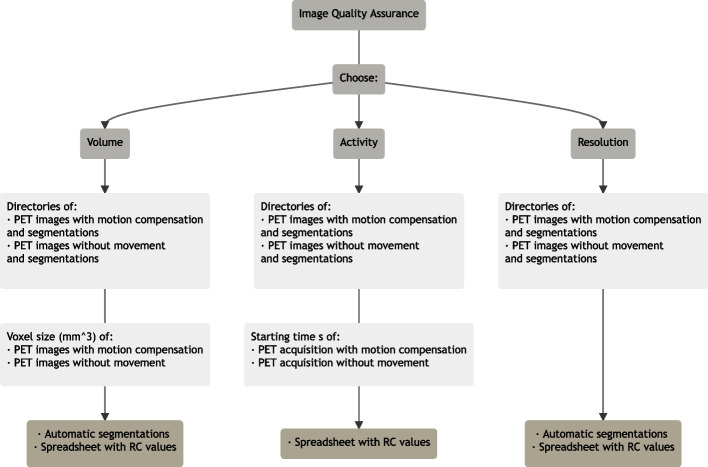
Scheme for *Image Quality Assurance* workflow of the open-source package. Light gray boxes indicate the inputs necessary for each type of quantification analysis. Brown boxes indicate the outputs of each analysis

We developed an open-source package in Python programming language [32] to perform the analysis described above. The package was divided into two sections, *Register and resampling* and *Image Quality Assurance*, being freely shared under the GNU General Public License in the repository *Quality Assurance* (https://github.com/AndreaMovilla/Quality-Assurance). The input data required from the user were: the images corresponding to the ideal response, that in our case were the static reference images of the 3 phantoms without movement; the set of N 3D images to be compared, that in our case were the images derived from the 10 different motion compensation protocols and one set of 6 manual segmentations for each analysis. The scheme of *Image Quality Assurance* is presented in Fig. 6. Detailed description can be found in the documentation of the package.

The homemade package provided RC values for VA, CA and R of 102 PET images in less than 5 min. The scripts’ performance has been validated with the open-source software Medical Imaging Toolkit (MITK) [33].

### Comparison of protocols with motion compensation of Gemini TF-64 PET/CT

#### Volume

**Fig. 7:**
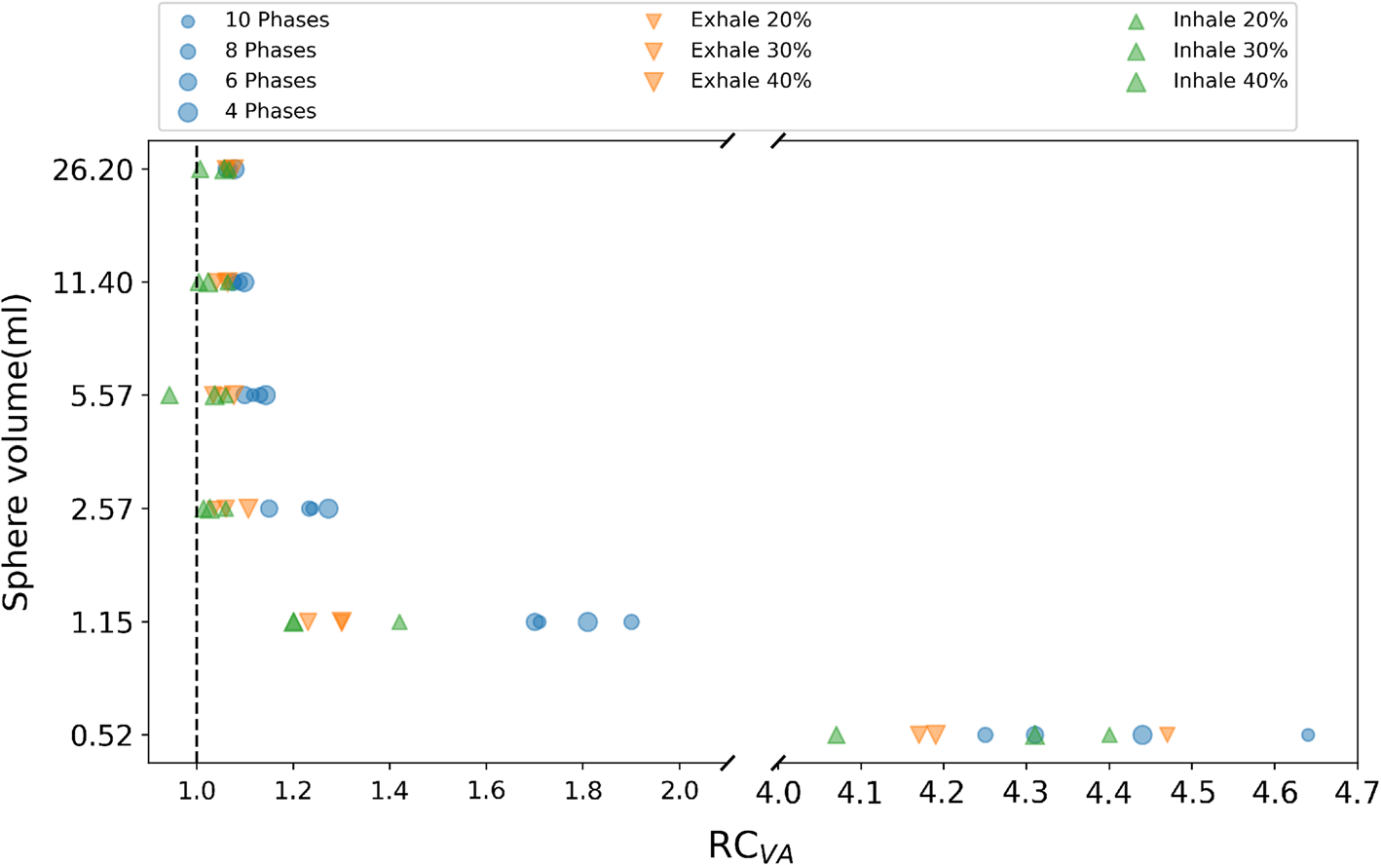
Average of the volume RC for the three movements per protocol for each NEMA sphere. RC is 1 for ideal response

**Fig. 8:**
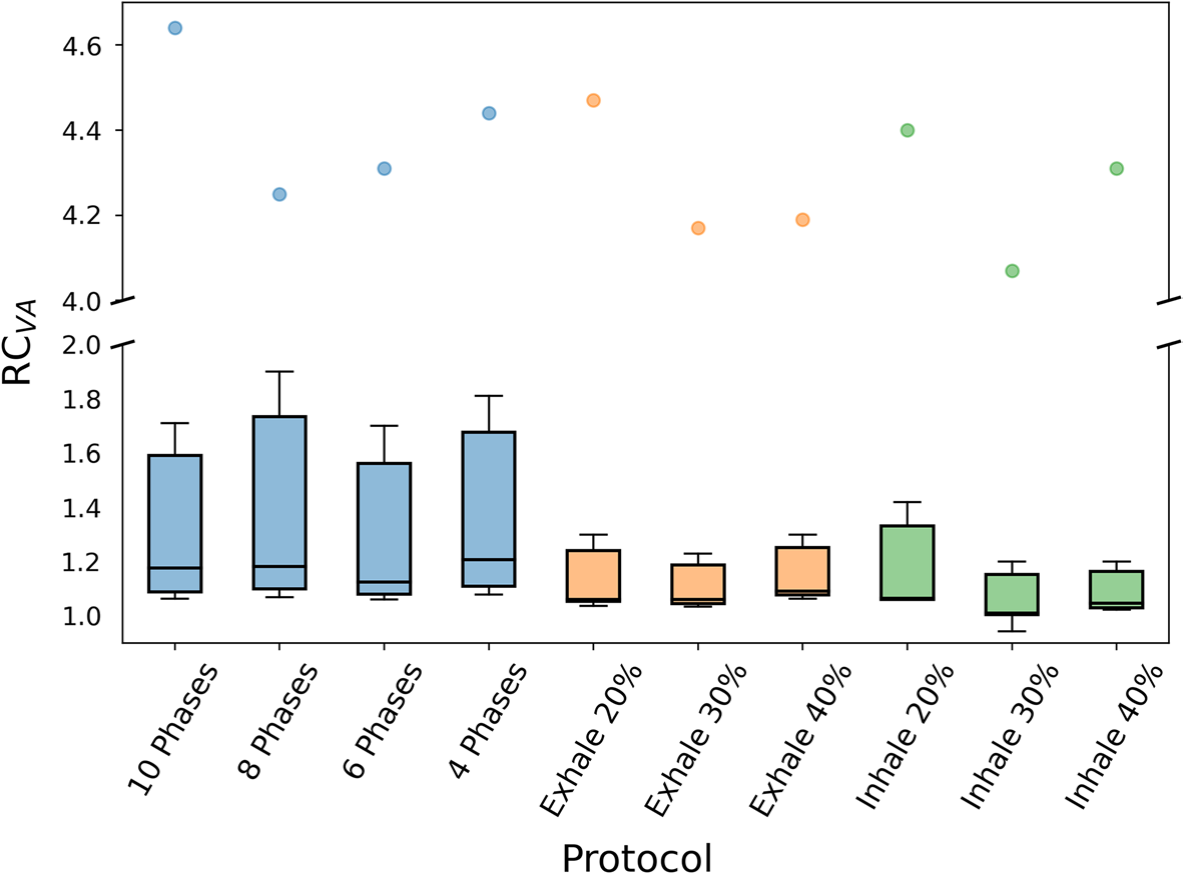
Boxplots of the volume RC per protocol. Each box represents the distribution of the 50% of the data. The whiskers represent the upper and lower quartiles. The bar color scheme represents the different types of protocols. Retrospective protocols are depicted in blue, while prospective Exhale and Inhale protocols are colored orange and green, respectively

For each of the 3 breathing patterns applied to the NEMA phantom, 34 images resulted from 10 protocols of motion compensation (4 retrospective and 6 prospective) were evaluated. For each image, the recovery coefficients with respect to the volumes estimated on the static image were calculated for the 6 spheres. Due to partial volume effect [34], for motion compensation protocols with poorest image quality, we observed failures in the segmentation of the smallest spheres. For these cases, RC was set to 5 as common indicator of the volume overestimation. RC averaged over the 3 different patterns is shown in Fig. 7. For retrospective protocols, the mean over the breathing phases was considered. In addition, boxplots are shown in Fig. 8.

As expected due to the partial volume effect, overestimation is more significant for smaller spheres, see in Fig. 6 the average values for small spheres and in Fig. 7 the outliers observed for all protocols, corresponding to the sphere of 0.52 ml. In general, better volume quantification was obtained for prospective protocols. In Fig. 7, we observed for prospective protocols more symmetric distributions, less data dispersion and medians closer to 1. Based additionally on the mean value of the RC over all the spheres ($1.5\pm 1.2$), Inhale 30% showed the best performance for volume quantification. From the retrospective protocols, 6 Phases showed the best performance with an averaged RC value over all the spheres of $1.7\pm 1.3$.

**Fig. 9:**
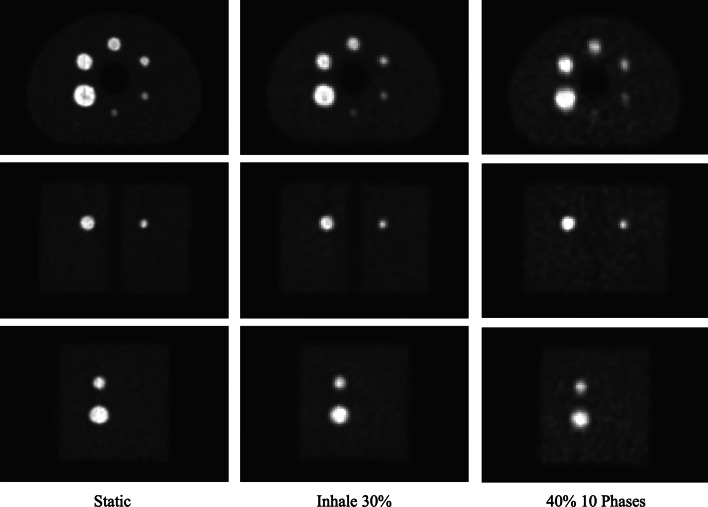
Coronal, axial and sagittal PET images for reference static protocol, Inhale 30% protocol and 10 Phases protocol of the NEMA phantom

In Fig. 9, the static PET image, the Inhale 30% PET image and the 40% PET image from the 10 Phases protocol are presented. By visual inspection, we could confirm that our results quantify image quality accordingly: Spread of the activity distribution in the spheres of the image obtained with protocol Inhale 30% is less significant than for the image obtained with protocol of 10 Phases, which also showed the poorest volume accuracy in terms of RC.

#### Activity concentration

**Fig. 10:**
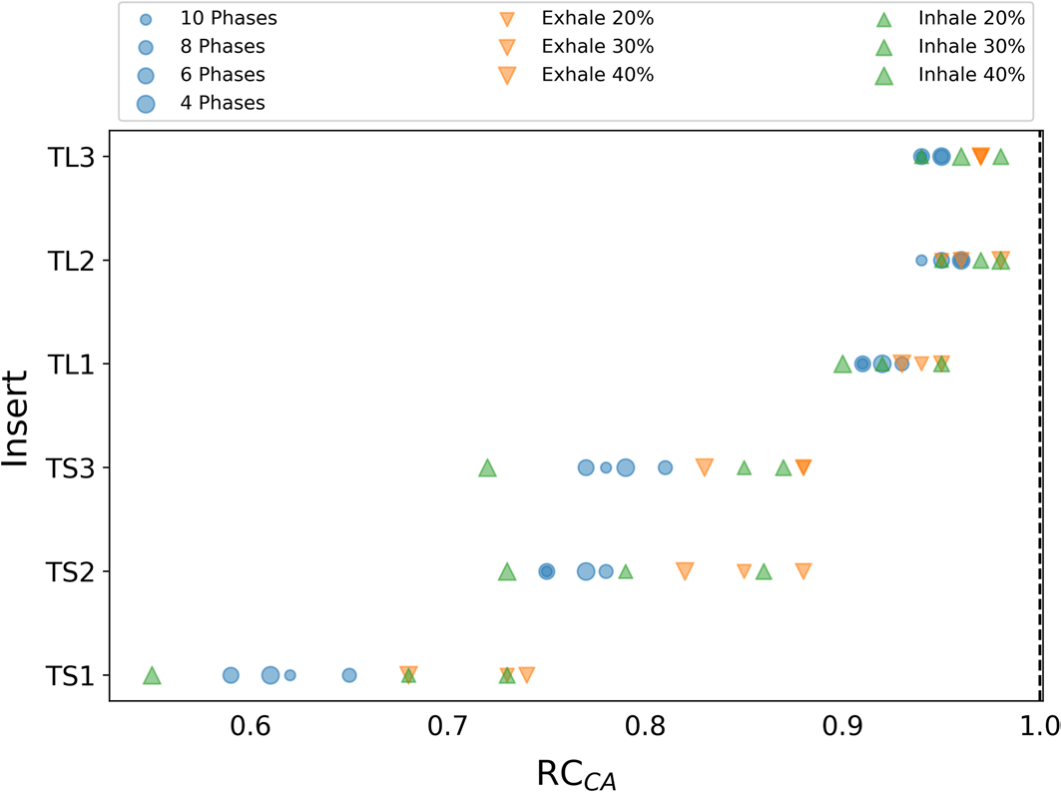
Average of the activity concentration RC of the three movements for each lesion

For each image with motion compensation, the recovery coefficients for the activity concentration of the six inserts within CIRS electron density phantom were calculated with respect to the static image. Results are shown in Fig. 10. As expected, quantification improves for large inserts ($\overline{RC}_{TL}=0.95$, $\overline{RC}_{TS}=0.76$).

**Fig. 11:**
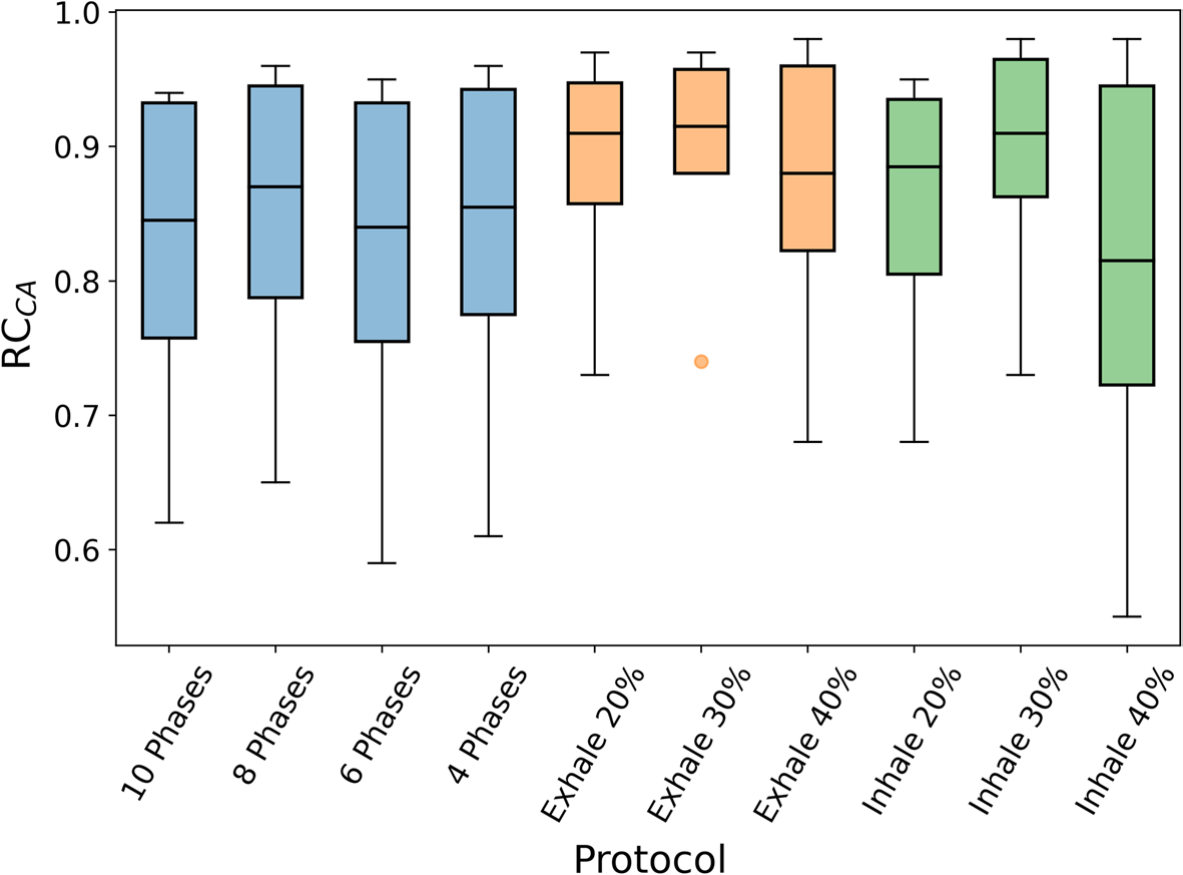
Boxplots of the activity concentration RC per protocol

Figure 11 shows the boxplots of the average of the RC for each protocol. In general, prospective protocols quantify activity concentration better than retrospective protocols. The protocols with less dispersion and medians closer to 1 are Exhale 30%, Exhale 20% and Inhale 30%. Based on mean RC, the best protocol for activity quantification is Exhale 30% ($0.90\pm 0.09$). For retrospective protocols, 8 Phases showed the best performance with $\overline{RC}=0.85 \pm 0.12$.

**Fig. 12:**
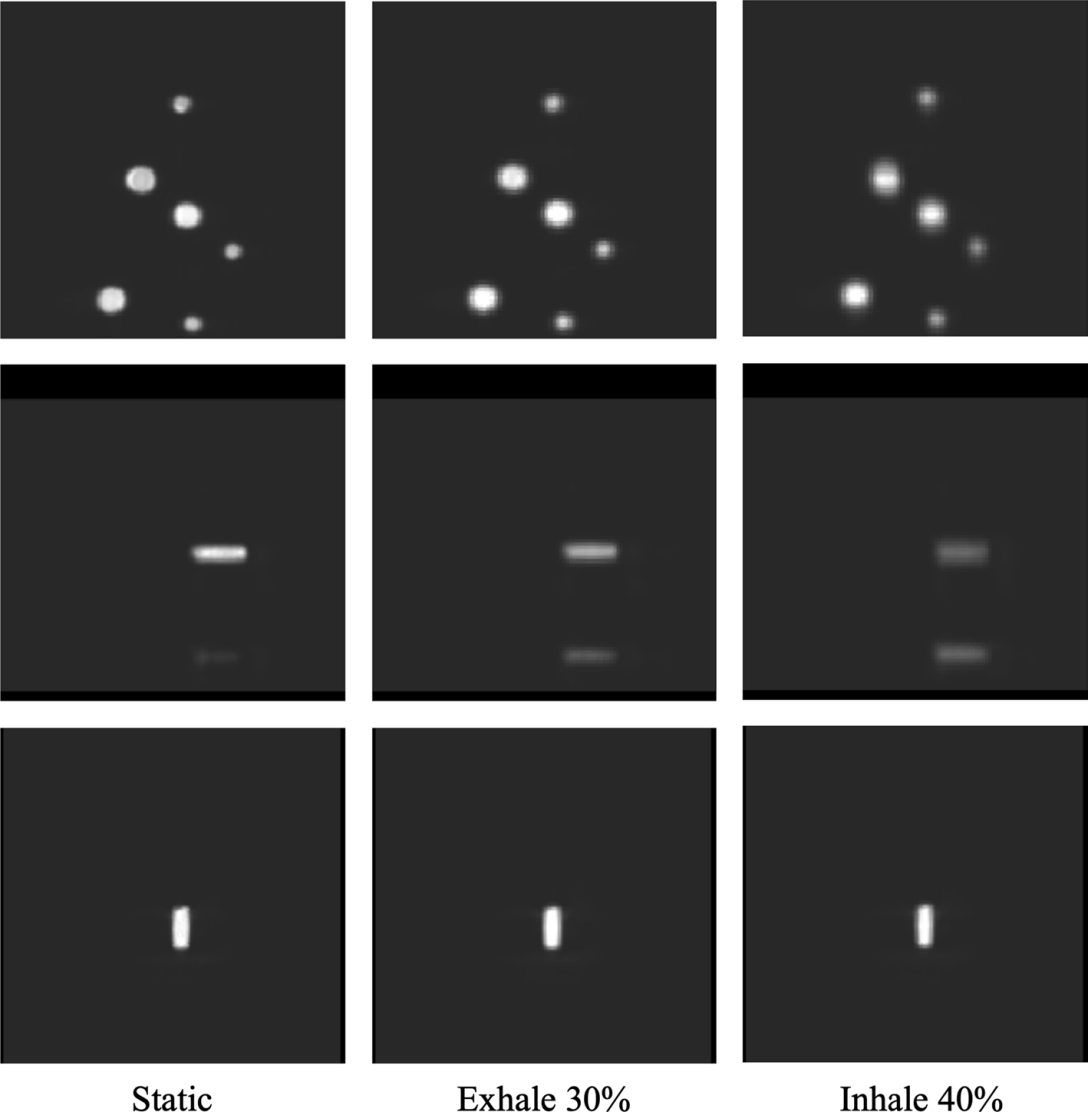
Coronal, axial and sagittal PET images for reference static protocol, Exhale 30% protocol and Inhale 40% protocol of the CIRS phantom

PET images for the static protocol, Exhale 30% protocol and Inhale 40% protocol are shown in Fig. 12. We qualitatively observed the poorer accuracy in concentration estimation for Inhale 40% with respect to Exhale 30%.

#### Spatial resolution

For each of the 6 sectors in the Jaszczak phantom, the number of rods observed on PET image is presented in Table 2. If we were able to identify at least 75% of the rods within the sector by applying the 40% segmentation, this sector was consider distinguished. Only the sector with biggest diameter, 12.7 mm, was distinguished in all protocols. Protocols Exhale 20%, Exhale 30% and Inhale 30% permitted additionally to distinguish sector with 11.1 mm of rod diameter. For sectors with rod diameters of 4.8 mm and 6.4 mm, segmentation failure was observed in all PET images, even in static PET protocol. For the rest of the spatial resolution evaluation, only the 4 sectors with larger diameters have been further analyzed.

**Table 2:** Number of rods distinguished per protocol and per rod sector

Rod diameter (mm)	4.8	6.4	7.9	9.5	11.1	12.7	Rods	Sectors
Total number in phantom	56	32	21	15	10	8	143	6
Reference: static	1	8	21	15	10	8	63	4
10 Phases	X	X	3	6	7	7	22	1
8 Phases	X	X	2	5	5	8	21	1
6 Phases	X	0	2	4	5	7	18	1
4 Phases	X	X	2	4	4	7	16	1
Exhale 20%	X	0	1	7	9	8	25	2
Exhale 30%	X	X	2	6	9	9	26	2
Exhale 40%	X	0	1	3	7	9	19	1
Inhale 20%	X	X	2	4	7	8	21	1
Inhale 30%	X	X	3	4	9	8	24	2
Inhale 40%	X	X	3	7	7	7	25	1

Segmentation failure marked with an X

**Fig. 13:**
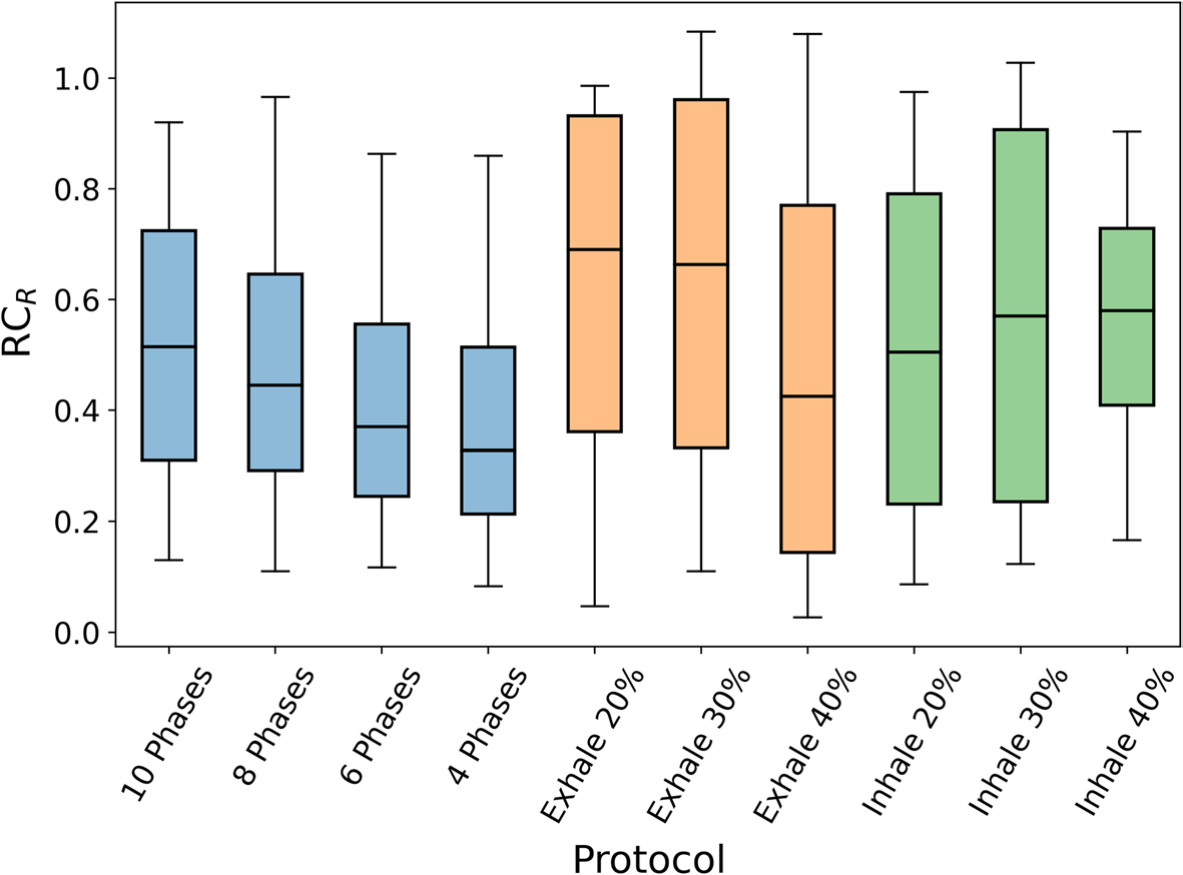
Boxplots of the rod RC per protocol

In Fig. 13, the boxplots of rod recovery coefficient for each protocol are presented. In prospective protocols, median values were closer to one. From the three protocols able to distinguish two sectors (Exhale 20%, Exhale 30% and Inhale 40%), Exhale 30% showed the best performance with the largest number of total rods (26) and the best RC mean ($0.6\pm 0.4$). For retrospective protocols, 10 Phases showed the best performance with an averaged RC of $0.5\pm 0.3$.

**Fig. 14:**
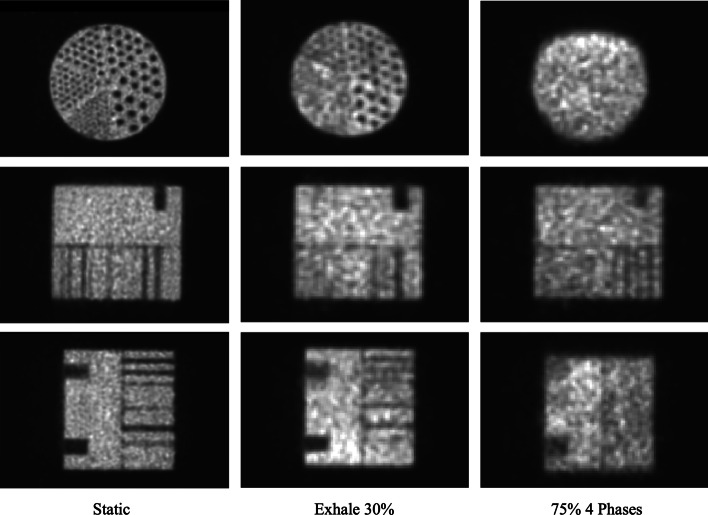
Coronal, axial and sagittal PET images for reference static protocol, Exhale 30% protocol and 4 Phases protocol of the Jaszczak phantom

In Fig. 14, static PET image, Exhale 30% image and the image of 75% from 4 Phases protocol are displayed. In agreement with our RC results, while in the static image four sectors were clearly visible, in the Exhale 30% image the two biggest sectors were distinguishable to the naked eye and no sectors were recognizable in the image chosen from the 4 Phases reconstruction.

### Comparison

**Fig. 15:**
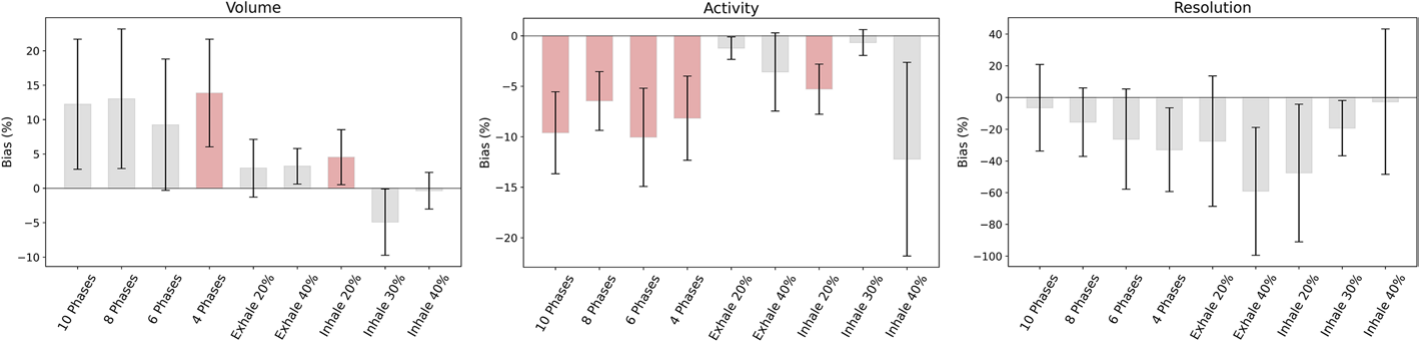
Bland–Altman and WSRT results for the comparison of Exhale 30% with respect to all protocols. The columns represent the average value for the relative deviation in percentage between paired series of data. The error bars represent the interval of confidence. When the interval involves the zero, series are considered comparable. The protocols not comparable under WSRT are shown in red

**Fig. 16:**
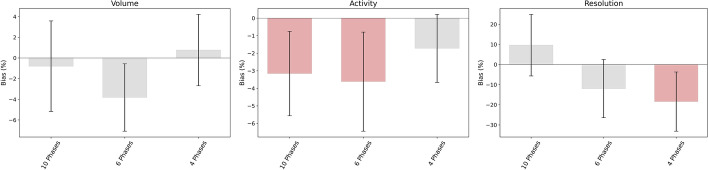
Bland–Altman and WSRT results for the comparison of 8 Phases with respect to retrospective protocols. The columns represent the average value for the relative deviation in percentage between paired series of data. The error bars represent the interval of confidence. When the interval involves the zero, series are considered comparable. The protocols not comparable under WSRT are shown in red

From the results obtained for each type of quantification analysis, we could conclude that the best performance was obtained for protocol Exhale 30%. From the retrospective protocols, 8 Phases showed the best performance. Performance for Exhale 30% was compared, in terms of BA analysis and WSRT, with respect to performance obtained for the other 9 protocols (Fig. 15). Inhale 30% showed a performance comparable to Exhale 30%, with compatible BA analysis results and WSRT (p$>0.05/10$, Bonferroni correction), over all three image quality parameters. In addition, 8 Phases were compared with respect to the other 3 retrospective protocols (Fig. 16). No one of the retrospective protocols was comparable with respect to 8 Phases.

Moreover, as an indicator of image noise of the protocols with the best performance, average value of the noise for a 30ml volume within the background of NEMA phantom has been averaged over all respiratory patterns. Image noise was 1404.33 Bq/ml for Exhale 30% and 1774.84 Bq/ml for 8 Phases.

### General applicability to other phantoms and scanners

As a proof of the general applicability of the proposed method to other phantoms and scanners, we applied the activity concentration analysis to other system, SIGNA PET/MR, and to other phantom, QUASAR$^\mathrm{{TM}}$ MRI$^{4D}$ Motion Phantom. We evaluated different prospective motion compensation protocols, with (trigger) and without (no trigger) rejection of irregular breathing cycles.

Based on the results for RC$_{CA}$ averaged over the 4 breathing patterns, the best protocol for activity quantification was Exhale 15% (trigger) with $\overline{RC}_{CA}=0.83 \pm 0.16$. However, the BA plot analysis showed that the performance of the protocols in the absence and presence of trigger rejection was comparable.

**Fig. 17:**
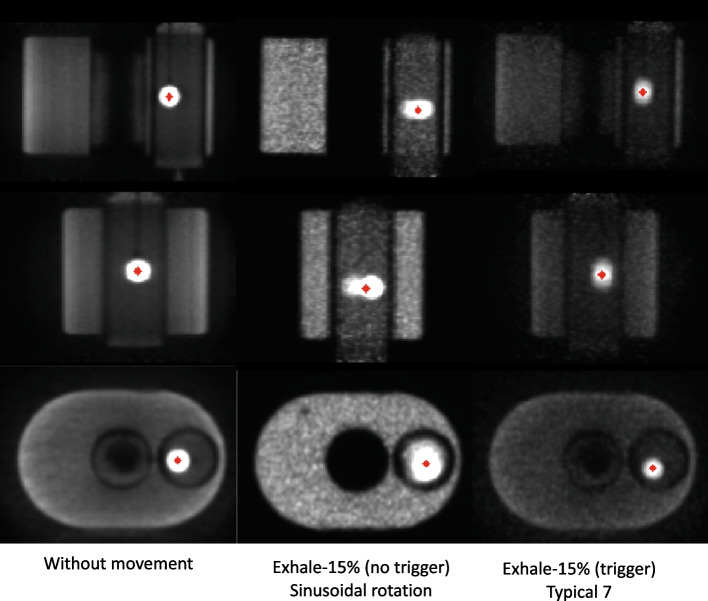
Coronal, sagittal and axial PET images for reference static protocol and the Exhale 15% (no trigger) for the Sinusoidal pattern with rotation and the Exhale 15% (trigger) for pattern Typical 7. Reference segmentations are shown in red

As was the case for the PET/CT experiment, results for RC$_{CA}$ in the PET/MR experiment also described quantitatively what was observed qualitatively. For example, a more significant degradation in the quality of PET image was observed by Exhale 15% (no trigger) for the Sinusoidal pattern with rotation than by Exhale 15% (trigger) for the Typical 7 pattern (Fig. 17) and accordingly, RC$_{CA}$ values were 0.6 and 0.94, respectively.

## Discussion

From our knowledge, we provided the first open-source package for the comparison of respiratory motion compensation PET/CT protocols. The comparison of protocols is based on the evaluation of PET image quality in terms of volume, activity concentration and spatial resolution. With this purpose, a specific and easily reproducible experimental phantom setup is presented. The proposed method has been applied for the comparison of the respiratory motion compensation protocols available in the Philips Gemini TF-64 PET/CT. Additionally, it could be employed with other phantoms and to evaluate other motion compensation protocols.

Even though 4D-PET/CT acquisition has been proven useful in the clinical setting, its use is not standardized. Frood et al. [15] review literature surrounding the use of 4D-PET/CT in pulmonary lesion characterization and conclude that PET/CT gating is underutilized because of practical limitations in hardware gating and lack of standardization of newer techniques. To promote the use of 4D-PET/CT gating, the analysis proposed in this work facilitates 4D protocol optimization for PET/CT systems by allowing the comparison and validation of different techniques across different vendors or research groups. The development and sharing of an open-source package aimed to simplify the validation and standardization of PET protocols. Its automatic nature could facilitate its application. In addition, our evaluation was based on experimental phantom measurements. The phantoms used for our analysis are usually available in nuclear medicine (NEMA IEC body phantom, Jaszczak phantom) and radiotherapy departments (electron density phantom for CT). It therefore facilitates the reproducibility of our study and the comparison between systems across different institutions. Additionally, in contrast to other commercially available packages for PET quality control, our software could be applied to other phantoms. The only requisite is to perform the reference segmentations consequently. However, we would like to remark that the results of the analysis may be meaningless if the phantoms used do not meet specific characteristics: Phantoms with different volumes are desirable for the evaluation of volume accuracy, phantoms with different densities are recommendable for a proper evaluation of the impact of attenuation map on the accuracy in the estimation of activity concentration and phantoms with a proper spatial pattern should be involved in the evaluation of resolution.

In our study, PET image quality was evaluated in terms of volume, activity concentration and resolution, which allows the reader to refer to the specific results depending on the PET image clinical application. Additionally, volume results are separated for small spheres, with non-negligible partial volume effect $<3$FWHM [34] and large spheres, allowing reader decision making based on the range of lesion sizes involved in the patient cohort under evaluation.

From the movement compensation protocols provided by the Philips Gemini TF-64 PET/CT system, prospective protocols showed the best overall response. Prospective protocols are limited to acquire data for a single breathing cycle phase, which might not be an issue for diagnose, treatment monitoring or the development of predictive models. However, in some clinical application, as, for example, radiotherapy planning, the location of the lesion during the breathing cycle might be of interest [35]. In this case, from our results we recommend the protocol with 8 Phases. The results reported in this study are specific to the protocols of motion compensation implemented in the Philips Gemini TF-64 PET/CT system. These protocols employed the time-based approach, which is the most common approach in the commercially available PET/CT systems: 3 systems of Philips (Gemini, Ingenuity and Vereos), 10 of GE (Discovery RX, Discovery 600, ..., Discovery Mi DR), the Celestion PET/CT of Canon, etc. Consequently, our results are of interest for users of a large number of PET/CT systems available on the market. In addition, other PET/CT system vendors or developers of motion compensation algorithms could reproduce our analysis and compare their performance with respect to our results. The proposed setup and open-source package have been successfully used with a different phantom and hybrid system, showing that the applicability of the proposed framework is independent of the phantom, system and protocols presented on this study. The results derived from the PET/MR experiment could be used to justify the use of movement compensation protocols without trigger rejection.

Previous studies compared 3D-PET/CT acquisition with respiratory-gated PET/CT [4, 6, 12, 13, 36, 37]. However, none of them provided open-source packages which would allow analysis replication. Most of them were based on clinical data without ground truth available, the ones based on experimental phantom measurements simulated the respiratory movement with the simplest approach of a sinusoidal movement without hysteresis, they did not evaluate all the image quality parameters and no different tissue densities were considered when concentration accuracy was evaluated. Park et al. [6] evaluated volume RC for NEMA spheres following a sinusoidal movement as function of the number of phases (2, 5 10 and 20) considered for the retrospectively gated protocol. In agreement with our work, they rejected retrospectively gating with 10 and more phases for large overestimation of the volume. 5 Phases showed the most accurate volume estimation, being 8 Phases (the protocol with the best volume accuracy in our study) not evaluated.

In contrast to previous publications [6, 18, 38, 39], in our study realistic breathing patterns representative of 60% of the population have been applied [25]. The breathing patterns chosen for our study had large amplitudes, as it has been proven that they have a significant impact on image quality [40]. In addition, we simulated hysteresis displacement [26]. A limitation of the study is the lack of atypical high irregular breathing patterns. However, in clinical practice, 4D acquisitions are usually preceded by a respiratory training and monitoring devices, in order to help the patient to follow a regular breathing. Another limitation was that the respiratory pattern was applied to the whole phantom. Consequently, the deformation effect observed in the organs during breathing was not simulated. The degradation of PET image quality due to this non-rigid movement is expected to be mainly due to attenuation map inaccuracies and with an impact that would not be as significant as the simulated by our CIRS phantom setup. The use of digital phantoms such as 4D-MCAT, 4D-XCAT and 4D-CAT [41], mimicking changes of lung and displacement of abdominal organs, could be recommended as an external validation of the results.

## Conclusion

We present an effective and reproducible methodology for comparing PET/CT movement correction protocols based on image quality. We additionally provide an open-source package. The feasibility of the proposed method has been proved by the comparison of the protocols available in the Philips Gemini TF-64 PET/CT. From the result of this comparison, Exhale 30% should be recommended.

## Data Availability

The dataset analyzed during the current study is available from the corresponding author on reasonable request.

## Abbreviations

PET: Positron emission tomography
CT: Computed tomography
$$^{18}$$F-FDG: $$^{18}$$F-fluorodeoxyglucose
SUV: Standardized uptake value
RC: Recovery coefficient
VA: Volume accuracy
CA: Activity concentration accuracy
R: Spatial resolution
BA: Bland–Altman
WSRT: Wilcoxon signed rank test
CI: Confidence interval
FWHM: Full width at half maximum
3D-PET/CT: Three-dimensional PET/CT
4D-PET/CT: Four-dimensional PET/CT

## References

[1] van Tinteren H, Hoekstra OS, Smit EF, van den Bergh JHAM, Schreurs AJM, Stallaert RALM (2002). Effectiveness of positron emission tomography in the preoperative assessment of patients with suspected non-small-cell lung cancer: the PLUS multicentre randomised trial. Lancet (London, England).

[2] Pommier P, Touboul E, Chabaud S, Dussart S, Pechoux CL, Giammarile F (2010). Impact of (18)F-FDG PET on treatment strategy and 3D radiotherapy planning in non-small cell lung cancer: A prospective multicenter study. AJR Am J Roentgenol.

[3] Guralnik L, Rozenberg R, Frenkel A, Israel O, Keidar Z (2015). Metabolic PET/CT guided lung lesion biopsies: impact on diagnostic accuracy and rate of sampling error. J Nucl Med.

[4] Callahan J, Kron T, Siva S, Simoens N, Edgar A, Everitt S (2014). Geographic miss of lung tumours due to respiratory motion: a comparison of 3D vs 4D PET/CT defined target volumes. Radiat Oncol (Lond, Engl).

[5] Büther F, Vehren T, Schäfers KP, Schäfers M (2016). Impact of data-driven respiratory gating in clinical PET. Radiology.

[6] Park SJ, Ionascu D, Killoran J, Mamede M, Gerbaudo VH, Chin L (2008). Evaluation of the combined effects of target size, respiratory motion and background activity on 3D and 4D PET/CT images. Phys Med Biol.

[7] Dawood M, Büther F, Lang N, Schober O, Schäfers KP (2007). Respiratory gating in positron emission tomography: a quantitative comparison of different gating schemes. Med Phys.

[8] Kruis MF, van de Kamer JB, Vogel WV, Belderbos JS, Sonke JJ, van Herk M (2015). Clinical evaluation of respiration-induced attenuation uncertainties in pulmonary 3D PET/CT. EJNMMI Phys.

[9] Oncofreeze AI. Accessed 8 June 2022. https://www.siemens-healthineers.com/molecular-imaging/options-and-upgrades/software-applications/oncofreeze.

[10] MotionFree. Accessed 8 June 2022. https://www.gehealthcare.com/products/molecular-imaging/pet-ct/motion-free.

[11] Kesner AL, Chung JH, Lind KE, Kwak JJ, Lynch D, Burckhardt D (2016). Validation of software gating: a practical technology for respiratory motion correction in PET. Radiology.

[12] Nehmeh SA, Erdi YE, Ling CC, Rosenzweig KE, Schoder H, Larson SM (2002). Effect of respiratory gating on quantifying PET images of lung cancer. J Nucl Med.

[13] Sindoni A, Minutoli F, Pontoriero A, Iatì G, Baldari S, Pergolizzi S (2016). Usefulness of four dimensional (4D) PET/CT imaging in the evaluation of thoracic lesions and in radiotherapy planning: Review of the literature. Lung Cancer.

[14] Didierlaurent D, Ribes S, Batatia H, Jaudet C, Dierickx LO, Zerdoud S (2012). The retrospective binning method improves the consistency of phase binning in respiratory-gated PET/CT. Phys Med Biol.

[15] Frood R, McDermott G, Scarsbrook A (2018). Respiratory-gated PET/CT for pulmonary lesion characterisation-promises and problems. Br J Radiol.

[16] Huang TC, Chou KT, Wang YC, Zhang G. Motion freeze for respiration motion correction in PET/CT: a preliminary investigation with lung cancer patient data. BioMed Res Int. 2014;2014. 10.1155/2014/167491 PMC4164623 25250313

[17] Li T, Zhang M, Qi W, Asma E, Qi J (2020). Motion correction of respiratory-gated PET images using deep learning based image registration framework. Phys Med Biol.

[18] Chang G, Chang T, Pan T, Clark JW, Mawlawi OR (2010). Joint correction of respiratory motion artifact and partial volume effect in lung/thoracic PET/CT imaging. Med Phys.

[19] DiFilippo FP, Patel M, Patel S (2019). Automated quantitative analysis of American College of Radiology PET phantom images. J Nucl Med Technol.

[20] Phantom Analysis Toolkit. Accessed 8 June 2022. https://www.snmmi.org/PAT.

[21] Zukić D, Byrd DW, Kinahan PE, Enquobahrie A (2018). Calibration software for quantitative PET/CT imaging using pocket phantoms. Tomography.

[22] Ulrich EJ, Sunderland JJ, Smith BJ, Mohiuddin I, Parkhurst J, Plichta KA (2018). Automated model-based quantitative analysis of phantoms with spherical inserts in FDG PET scans. Med Phys.

[23] QUASAR Respiratory Motion Platform.

[24] QUASAR MRI 4D Motion Phantom. Accessed 12 Sept 2022. https://modusqa.com/products/quasar-mri4d-motion-phantom/.

[25] Liu C, Pierce LA, Alessio AM, Kinahan PE (2009). The impact of respiratory motion on tumor quantification and delineation in static PET/CT imaging. Phys Med Biol.

[26] Seppenwoolde Y, Shirato H, Kitamura K, Shimizu S, van Herk M, Lebesque JV (2002). Precise and real-time measurement of 3D tumor motion in lung due to breathing and heartbeat, measured during radiotherapy. Int J Radiat Oncol Biol Phys.

[27] Surti S, Kuhn A, Werner ME, Perkins AE, Kolthammer J, Karp JS (2007). Performance of Philips Gemini TF PET/CT scanner with special consideration for its time-of-flight imaging capabilities. J Nucl Med.

[28] Grant AM, Deller TW, Khalighi MM, Maramraju SH, Delso G, Levin CS (2016). NEMA NU 2–2012 performance studies for the SiPM-based ToF-PET component of the GE SIGNA PET/MR system. Med Phys.

[29] Zaidi H, El Naqa I (2010). PET-guided delineation of radiation therapy treatment volumes: a survey of image segmentation techniques. Eur J Nucl Med Mol Imaging.

[30] Adams R, Bischof L (1994). Seeded region growing. IEEE Trans Pattern Anal Mach Intell.

[31] Giavarina D (2015). Understanding Bland Altman analysis. Biochem Med.

[32] Python Software Foundation. Accessed 8 Feb 2022. https://www.python.org/.

[33] Wolf I, Vetter M, Wegner I, Böttger T, Nolden M, Schöbinger M (2005). The medical imaging interaction toolkit. Med Image Anal.

[34] Soret M, Bacharach SL, Buvat I (2007). Partial-volume effect in PET tumor imaging. J Nucl Med.

[35] Bowen SR, Nyflot MJ, Gensheimer M, Hendrickson KRG, Kinahan PE, Sandison GA (2012). Challenges and opportunities in patient-specific, motion-managed and PET/CT-guided radiation therapy of lung cancer: review and perspective. Clin Transl Med.

[36] Bailly P, Bouzerar R, Shields T, Meyer ME, Daouk J (2018). Benefits of respiratory-gated 18F-FDG PET acquisition in lung disease. Nucl Med Commun.

[37] Watanabe S, Hanaoka K, Kaida H, Hyodo T, Yamada M, Tsurusaki M (2021). Usefulness of respiratory-gated PET acquisition during delayed 18F-FDG PET/CT scanning for patients with liver metastases. Asia Ocean J Nucl Med Biol.

[38] Callahan J, Kron T, Schneider-Kolsky M, Dunn L, Thompson M, Siva S (2013). Validation of a 4D-PET maximum intensity projection for delineation of an internal target volume. Int J Radiat Oncol Biol Phys.

[39] Nehmeh SA, Erdi YE, Rosenzweig KE, Schoder H, Larson SM, Squire OD (2003). Reduction of respiratory motion artifacts in PET imaging of lung cancer by respiratory correlated dynamic PET: methodology and comparison with respiratory gated PET. J Nucl Med.

[40] Ionascu D, Jiang SB, Nishioka S, Shirato H, Berbeco RI (2007). Internal-external correlation investigations of respiratory induced motion of lung tumors. Med Phys.

[41] Segars WP, Tsui BMW (2009). MCAT to XCAT: the evolution of 4-D computerized phantoms for imaging research. Proc IEEE.

